# Flavor-protein interactions for four plant proteins with ketones and esters

**DOI:** 10.1016/j.heliyon.2023.e16503

**Published:** 2023-05-25

**Authors:** Silvia J.E. Snel, Mirela Pascu, Igor Bodnár, Shane Avison, Atze Jan van der Goot, Michael Beyrer

**Affiliations:** a Institute of Life Technologies, University of Applied Sciences and Arts Western Switzerland, CH-1950 Sion, Switzerland; b Food Process Engineering, Agrotechnology and Food Sciences Group, Wageningen University & Research, Bornse Weilanden 9, 6708 WG Wageningen, the Netherlands; c Firmenich S.A., Rue de la Bergère 7, Meyrin 2, CH-1217 Geneva, Switzerland

**Keywords:** Partitioning, Pea, Soy, Chickpea, Fava bean, Whey, Hydrophobic interactions

## Abstract

The interaction between flavors and proteins results in a reduced headspace concentration of the flavor, affecting flavor perception. We analyzed the retention of a series of esters and ketones with different chain lengths (C4, C6, C8, and C10) by protein isolates of yellow pea, soy, fava bean, and chickpea, with whey as a reference. An increase in protein concentration led to a decrease in flavor compound in the headspace as measured with atmospheric pressure chemical ionization time-of-flight mass spectroscopy (APCI-TOF-MS). Flavor retention was described with a flavor-partitioning model. It was found that flavor retention could be well predicted with the octanol-water partitioning coefficient and by fitting the hydrophobic interaction parameter. Hydrophobic interactions were highest for chickpea, followed by pea, fava bean, whey, and soy. However, the obtained predictive model was less appropriate for methyl decanoate, possibly due to its solubility. The obtained models and fitted parameters are relevant when designing flavored products with high protein concentrations.

## Highlights

• Retention of esters and ketones by four plant proteins and whey was measured.
• An increase in chain length resulted in increased retention of esters and ketones.
• Retention could be explained assuming solely hydrophobic interactions.
• A hydrophobic interaction parameter was fitted and followed: chickpea > pea > fava bean > whey > soy.
• The hydrophobic interaction parameter was correlated with the hydrophobic index of plant proteins.

## 1 Introduction

The replacement of animal products, such as meat and dairy, with plant-based alternatives could help to reduce the impact of diet choices on the environment [1], [14], [22]. Nevertheless, the flavor of these products, such as plant-based meat, is considered less attractive and appealing to consumers who are not vegan or vegetarian [19]. Flavors are added to improve the hedonistic properties of meat and dairy replacers, by masking off-flavors and improving the overall flavor profile. The addition of flavors might not be straightforward, since these analog products often contain plant-based proteins to which flavors can strongly bind to or interact [10]. Important plant-based proteins for meat, dairy and egg analogs are soy, pea, and fava bean, due to their gelling and emulsifying ability [18], [15]. Protein-flavor interactions are mainly caused by reversible hydrophobic interactions, hydrophilic interactions, Van der Waal's forces, and ionic bonds, but can also be caused by irreversible covalent binding [25]. The degree of flavor retention depends on the protein source [23], the method of protein extraction [25], and the chemical class of the flavors [23], [25], [12], [9]. A common approach to study the protein-flavor interaction is to measure equilibrium headspace concentration and to express flavor retention as a percentage of reduced headspace concentration compared to the control [9], [28], [26]. However, this approach is time-consuming and therefore often only a few chemicals are studied, which makes it challenging to offer a mechanism behind the retention. An alternative method to study protein-flavor interactions is to model experimental data to predict flavor partitioning. Harrison and Hills developed a mathematical model to predict flavor retention for both hydrophilic and hydrophobic compounds from a liquid containing macromolecules [11], [11]. This model was applied to predict flavor-retention in casein/whey dispersions with esters, alcohols, and aldehydes [23]. The study showed that it was sufficient to assume hydrophobic interactions to explain flavor-retention for esters and alcohols [23]. Recently, partitioning coefficients have been calculated for pea with hexanal and 2-octenal [6]. However, to our knowledge, no attempt has been made to model flavor partitioning for plant-based proteins so far. A better understanding of the flavor retention in a range of leguminous proteins can help in targeted flavoring of meat analogues. It can be achieved by investigating the applicability of the partitioning model to plant-based proteins. The aim of this work is, therefore, to apply the flavor partitioning model to different plant-based proteins, important for the production of plant-based substitutes. The proteins studied are pea protein isolate (PPI), soy protein isolate (SPI), chickpea protein isolate (CPPI), and fava bean protein isolate (FBPI). Furthermore, whey protein isolate (WPI) will be included as a control, since the flavor retention to the main protein in whey, *β*-lactoglobulin, has been widely studied [5], [4], [3], [13]. The investigated flavors include a series (C4, C6, C8, C10) of ketones and esters. Ketones and esters are expected to interact non-covalently with proteins, making them suitable for the Harrison and Hills model. Furthermore, they can be added to meat analogues either as flavors or compounds that mask off-flavors. For each protein, 5 concentrations will be prepared and a control. This results in a large dataset of 240 variations in triplicate, which will be used for the model fitting.

## 2 Theory: flavor partitioning models

**Figure 1:**
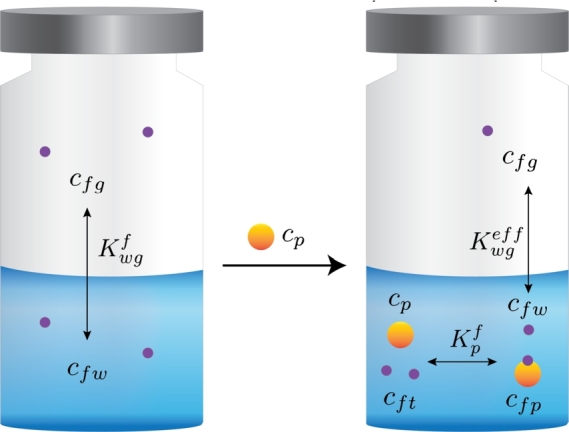
Simplified scheme of the flavor-water system (left), and flavored protein dispersion (right): *c* corresponds to concentrations of flavor in water (fw), flavor in the gas phase (fg), flavor bound to protein (fp), total flavor (ft) and protein (p). $K_{wg}^{f}$ is the partitioning coefficient between gas and water of the flavor, and ($K_{wg}^{eff}$) is the effective partitioning when protein is added. $K_{p}^{f}$ is the interaction constant between protein and flavor. Purple circles represent flavor molecules, and orange circles the protein.

Harrison and Hills [11] developed a mathematical model to predict flavor retention in an aqueous solution containing polymers. A system is considered that consists of a water (w) and gas (g) phase, where there is flavor bound to protein (fp), flavor in the water phase (fw), flavor in the gas phase (fg), and total flavor (ft) (Fig. 1). A partition coefficient $K_{wg}^{f}$ between flavor in the gas phase and the water phase is considered:

(1) $$K_{wg}^{f}=\frac{c_{fg}^{e}}{c_{fw}^{e}}$$

in which $c_{fg}^{e}$ and $c_{fw}^{e}$ are the concentration of flavor in the gas phase and the water phase respectively, at equilibrium conditions. However, some of the flavors in the water phase could interact with the protein. When the reversible first-order reaction is assumed for flavor-protein interactions, we obtain:

(2) $$PF\overset{K_{p}^{f}}{⇄}P+F$$

in which *PF*, *P*, and *F* represent the flavor retained by protein, protein, and flavor in the aqueous solution respectively. $K_{p}^{f}$ is the global interaction constant between protein P and flavor F. At equilibrium condition, $K_{p}^{f}$ becomes:

(3) $$K_{p}^{f}=\frac{c_{fp}^{e}}{c_{p}^{e}c_{fw}^{e}}$$

in which $c_{fp}^{e}$ and $c_{p}^{e}$ are the concentrations of protein-retained flavor in the dispersion at equilibrium, and protein. When the concentration of free protein exceeds the concentration of protein-flavor complexes largely, $c_{p}$ can be simply defined as the total protein concentration in the solution. Now, the effective partition coefficient $K_{wg}^{eff}$ between flavor in the gas phase and the water phase becomes:

(4) $$K_{wg}^{eff}=\frac{c_{fg}^{e}}{c_{ft}^{e}}$$

in which $c_{ft}^{e}$ is the total flavor in the water system. The simplified mass balance derived from eq (2) reads:

(5) $$c_{ft}=c_{fp}+c_{fw}$$

When no protein is present in the water phase, $c_{ft}$ is equal to $c_{fw}$. The mass balance (eq (5) is altered using eq (3) to obtain:

(6) $$c_{ft}=c_{fw}^{e}+K_{p}^{f}c_{p}^{e}c_{fw}^{e}=c_{fw}^{e}(1+K_{p}^{f}c_{p}^{e})$$

Eq (1) can be rewritten as a function of $c_{fg}$ and using eq (6) eq (4) becomes:

(7) $$K_{wg}^{eff}=\frac{c_{fw}^{e}K_{wg}^{f}}{c_{fw}^{e}(1+K_{p}^{f}c_{p}^{e})}$$

Eq (7) leads to:

(8) $$K_{wg}^{eff}=\frac{K_{wg}^{f}}{1+K_{p}^{f}c_{p}^{e}}$$

For ketones and esters, the flavor-protein interaction is assumed to be dominated by hydrophobic interactions, the global interaction constant could be approached with [23]:

(9) $$K_{p}^{f}=a_{p}P_{ow}^{f}$$

in which $a_{p}$ and $P_{ow}^{f}$ are the unknown hydrophobic interaction parameter of the protein and the known octanol-water partition coefficient of the flavor compound.

## 3 Methods and materials

### 3.1 Materials

Soy protein isolate (SPI, Supro® 500E A) was obtained from Solae (Europe S.A.). Pea protein isolate (PPI, Nutralys® F85M) was obtained from Roquette Frères S.A. (Lestrem, France). Fava bean protein isolate (FBPI, FFBP-90-C-EU) and chickpea protein isolate (CPPI, FCPP-70) were both obtained from AGT Foods (Regina, Canada). Whey protein isolate (WPI, BiPRO) was obtained from Davisco Foods International (Minnesota, USA). The moisture contents of the powders were measured with a moisture analyzer (Mettler Toledo, Ohio, United States). Moisture contents were 8.8± 0.0 g/100g (SPI), 8.1± 0.0 g/100g (PPI), 8.0± 0.1 g/100g (FBPI), 6.6± 0.1 g/100g (CPPI), and 5.2± 0.1 g/100g (WPI). Methyl decanoate, 2-hexanone, 2-decanone, and ethanol were purchased from Sigma-Aldrich (St. Louis, USA). The remaining flavors listed in Table 1 were provided by Firmenich S.A.

**Table 1:** Flavors studied with their corresponding chemical class, molecular weight (MW), solubility, octanol-water partition coefficient (Log P), and the total concentration in the flavored protein dispersions in this study, corresponding to *c*_*ft*_ (ppm).

Name	Class	MW (gmol^−1^)^1^	Solubility (mgkg^−1^)^1^	Log P^1^	Concentration (mgkg^−1^)
2-Butanone	Ketone	72.11	76100	0.26	5.53
2-Hexanone	Ketone	100.16	7745	1.24	5.84
2-Octanone	Ketone	128.22	884	2.22	0.58
2-Decanone	Ketone	156.27	46.4	3.20	0.55
Methyl butanoate	Ester	102.13	9120	1.36	0.21
Methyl hexanoate	Ester	130.19	926	2.34	0.27
Methyl octanoate	Ester	158.24	102	3.32	0.59
Methyl decanoate	Ester	186.30	8.8	4.30	0.18

1 Software EPIWEB v4.1, KOWWIN v1.68

### 3.2 Protein content and amino acid composition

The crude nitrogen contents of the isolates were determined using the Kjeldahl method in triplicates (AACC, method 46-10). The nitrogen conversion factor was calculated for each isolate with its exact amino composition, which was measured by Triskelion in duplicate (Utrecht, Netherlands). The amino acids were determined with the AOAC 2018.06 method. In this method, samples were first hydrolyzed using hydrochloric acid (HCl). Afterwards, the amino acids were derivatized with phenylisothiocyanate, and analyzed by high-performance liquid chromatography (HPLC). HPLC was performed in the reverse phase. The amount of each amino acid is quantified by comparing its peak area to that of a known standard. For each amino acid, the weight of nitrogen per g amino acid was calculated (gg^−1^), by counting the number of nitrogen molecules, calculating the molecular weight, and dividing this by the molecular weight of the amino acid. Then, for each amino acid, the weight per 1 g of protein (g amino acid/g protein) was multiplied by the weight of nitrogen for this amino acid (g nitrogen/g amino acid). Subsequently, the values for each amino acid were summed and 1 g protein was divided by the total nitrogen content to obtain the conversion factor.

### 3.3 Hydrophobic index

By dividing the total hydrophobic content by the total hydrophilic content a hydrophobic:hydrophilic ratio was obtained for each protein. However, the different amino acids differ in degree of hydrophobicity [20]. Therefore, the hydrophobic index was calculated, using the hydrophobicity scale [21]. The hydrophobic index can give a comprehensive representation of hydrophobicity and allows a good comparison between the different proteins based on their amino acid composition. Hydrophobic amino acids got a positive index (maximum 100), whereas hydrophilic amino acids had a negative index (maximum -100) and neutral ones got an index of 0. Then, the fraction of amino acid (gg^−1^ protein) was multiplied with the corresponding hydrophobic score and summed:

(10) $$\sum [F_{Alanine}S_{Alanine}...F_{Valine}S_{Valine}]$$

in which *F* and *S* correspond to the fraction and score of the amino acid. This resulted in a hydrophobic index for each protein.

### 3.4 Solubility protein isolates

Solubility of the protein isolates was measured in triplicate with a method reported by [7] with some minor alterations. A dispersion of 0.0125 gg^−1^ protein isolate in demineralized water was prepared and shaken overnight at 300 rpm at room temperature. Subsequently, the dispersions were centrifugated at 10,000 g at 21°C for 30 min. The weight of the supernatant and pellet were recorded and both were dried at 100°C for at least 12 h. The solubility was calculated as follows:

(11) $$Solubility=\frac{M_{dry-powder}-M_{dry-pellet}}{M_{dry-powder}}$$

in which $M_{dry-powder}$ is the overall weight of the isolate and $M_{dry-pellet}$ is the weight of the pellet after drying.

### 3.5 Preparation flavored protein dispersions

First, a stock solution for each protein isolate was made of 50 gkg^−1^ in demineralized water. At this step, the concentration of protein isolate represents total isolate content, not yet corrected for dry matter and protein content. Stock solutions were first stirred at 21°C for 1 h, then finely dispersed with an Ultra Turrax (T25, IKA) and subsequently left for complete hydration at 5°C for 24 h. Subsequently, either 0.5, 1, 2, 3, or 5 mL of the stock solution was transferred to crimp vials and demineralized water was added to obtain a total of 5 mL. This resulted in dispersions of 5, 10, 20, 30, and 50 gkg^−1^ for each protein isolate. The concentrations were chosen such that flavor retention was expected to occur, but retention was not maximum. The exact amount of protein in each dispersion was then calculated based on the protein content and dry matter content of the protein isolates. Each flavor compound was first diluted in ethanol, to ensure solubility, and then added to the protein dispersion to reach a concentration in a range from 0.1 to 10 mgkg^−1^(ppm). The concentration used depended on the flavor compound. The final concentrations of each flavor compound in the aqueous phase were chosen such that it was within the range of the calibration curve (Table 1). The flavored dispersions were then vortexed for 30 s. The presence of ethanol in the final solution (1 gkg^−1^) was tested and found to not affect the MS signal. The mixture was allowed to reach equilibrium at 21°C for 24 h.

### 3.6 Static headspace measurements

The headspace concentrations were measured at equilibrium as independent triplicates with a G2-XS Q-TOF high-definition mass spectrometer (XEVO, Waters) coupled to a patented Venturi interface [16]. An automated PAL system sampled 5 mL of headspace air with a 5 mL headspace syringe and injected this into the mass spectrometer. Mass spectra were collected in centroid mode over the range m/z 20-400 every 1 s. APCI-MS was performed in positive ionization mode with a cone voltage of 4.0 kV, source temperature of 105°C, heated sample transfer line temperature of 130°C and auxiliary gas flow of 600 Lh^−1^. Lock spray (on-the-fly mass calibration) was used to apply a mass correction to measured m/z values during the analysis. All the signal intensities were corrected for the background addition. The relative headspace concentration (RHC) was calculated as:

(12) $$\text{RHC}\text{\%}=\frac{\text{Peak area}{}_{\text{flavored dispersion}}}{\text{Peak area}{}_{\text{flavor in water}}}⁎100\text{\%}$$

### 3.7 Model fitting

The relative headspace concentration can be seen as $\frac{K_{wg}^{eff}}{K_{wg}^{f}}$ since the protein dispersion and water system are directly compared. Therefore, the exact concentration of retained flavor, free flavor, and total flavor are not needed in the model fitting. Eq (8) and (9) were combined and rewritten to obtain:

(13) $$\text{RHC}=\frac{K_{wg}^{eff}}{K_{wg}^{f}}=\frac{1}{1+a_{p}P_{ow}^{f}c_{p}}$$

For $K_{wg}^{f}$ we can assume that $c_{ft}=c_{fw}$. Thus, to simplify, $\frac{K_{wg}^{eff}}{K_{wg}^{f}}$ is interpreted as the concentration in the headspace with and without protein present in the water phase, and eq (13) is rewritten to obtain a linear relation:

(14) $$\frac{K_{wg}^{f}}{K_{wg}^{eff}}=\frac{c_{fg}}{c_{fg}^{p}}=1+a_{p}P_{ow}^{f}c_{p}$$

Eq (14) was fitted to the experimental results for ketones and esters to obtain the $a_{p}$ values. The slope of the linear lines is $a_{p}P_{ow}^{f}$, and thus a measure of binding. A higher slope means more binding per gram of protein. The calculated octanol-water partition coefficient was obtained from EPIWEB. The $c_{p}$ was taken as the concentration of protein, thus corrected with the dry matter and protein content for each isolate. The fitting was done with the complete dataset, rather than with the averages. The fitting parameter $a_{p}$ was fitted per protein for esters and per protein for ketones. Data that did not show a linear relation was excluded from the fits. The fitting was performed with Python and the SciPy package. This resulted in a prediction for $a_{p}$ and the uncertainty of this prediction.

### 3.8 Statistical analysis

Statistical analysis was performed with R. Normality was tested with descriptive statistics. When the data were normally distributed, a one-way analysis of variance (ANOVA) was done to test if the observed differences between samples were significant. Multiple comparison Tukey tests were done to indicate which treatments were significantly different from each other. A correlation matrix was obtained between measured parameters and fitting results.

## 4 Results

### 4.1 Chemical composition protein isolates

For PPI, SPI, FBPI, and CPPI the amino acid composition was measured (Table 2). With the obtained amino acid composition, the nitrogen conversion factor was calculated, and thereafter the protein content for each isolate with the obtained Kjeldahl results. SPI had the highest protein content of the plant proteins (83.25 g/100g), followed by FBPI (80.42 g/100g), PPI (77.11 g/100g), and CPPI (67.03 g/100g). The plant proteins were very close in their amino acid profile, as has also been observed previously for SPI, PPI, and FBPI [17]. The amino acid composition of WPI was not measured, but instead, we used values from literature in which a similar WPI was studied [2]. Reference values for WPI showed a clear difference in amino acid composition compared to the plant-based proteins. Subsequently, the hydrophobic:hydrophilic ratio and the hydrophobic index were calculated (eq (10)). WPI had a higher amount of total hydrophobic amino acids. Furthermore the hydrophobic:hydrophilic ratio and the hydrophobic index were also higher compared to the plant proteins. Within the plant proteins, CPPI had the highest hydrophobic index (15.23), followed by PPI (14.81), SPI (14.73), and FBPI (14.32). In addition, the pH, and solubility (eq (11)) of the isolates were measured for each isolate. PPI had the highest pH (7.5), followed by SPI and WPI (7.1), CPPI (6.6), and FBPI (6.4). WPI was completely soluble in water, whereas the plant proteins had a much lower solubility.

**Table 2:** Amino acid composition and the obtained nitrogen conversion factor and protein content, pH, and solubility of soy, pea, fava bean, chickpea, and whey protein isolates. Values are means ± standard deviations, letters indicate significant groups. Amino acids were measured in duplicate, and the protein content, pH, and solubility in triplicate. Hydrophobic indexes are a summation of the amino acid fraction multiplied by the hydrophobic score.

Amino acids (mg AA/g protein)		Soy	Yellow pea	Fava bean	Chickpea	Whey^1^
Hydrophilic	Arginine	78.03 ± 0.31^*d*^	89.24 ± 0.44^*c*^	98.56 ± 0.24^*a*^	93.77 ± 0.06^*b*^	26.2
	Asparagine +	113.98 ± 0.20^*c*^	118.00 ± 0.07^*b*^	114.02 ± 0.75^*c*^	123.27 ± 0.58^*a*^	121
	Aspartic acid					
	Glutamine +	192.06 ± 0.08^*a*^	174.31 ± 0.11^*c*^	181.47 ± 0.95^*b*^	173.56 ± 0.25^*c*^	175
	Glutamic acid					
	Histidine	27.34 ± 0.12^*a*^	26.13 ± 0.07^*b*^	27.44 ± 0.12^*a*^	26.49 ± 0.37^*b*^	19.1
	Lysine	62.39 ± 0.08^*c*^	75.80 ± 0.14^*a*^	65.70 ± 0.70^*b*^	64.93 ± 0.35^*b*^	116
	Serine	51.61 ± 0.17^*c*^	51.83 ± 0.10^*c*^	53.96 ± 0.36^*b*^	54.92 ± 0.06^*a*^	41.0
	Threonine	38.48 ± 0.14^*a*^	37.19 ± 0.05^*b*^	36.32 ± 0.20^*c*^	34.55 ± 0.14^*d*^	50.9
	Tyrosine	40.65 ± 0.04^*b*^	41.49 ± 0.08^*a*^	40.83 ± 0.29^*a*^*b*	33.17 ± 0.13^*c*^	39.8
	Total	604.55 ± 0.58^*c*^	613.99 ± 0.41^*b*^	618.29 ± 1.19^*a*^	604.66 ± 0.54^*c*^	589
Hydrophobic	Alanine	39.48 ± 0.02^*b*^	39.43 ± 0.05^*b*^	38.98 ± 0.16^*c*^	40.19 ± 0.01^*a*^	52.8
	Cysteine	12.26 ± 0.12^*a*^	10.15 ± 0.35^*b*^	8.39 ± 0.13^*c*^	10.74 ± 0.19^*b*^	32.4
	Glycine	36.28 ± 0.07^*a*^	35.87 ± 0.05^*a*^	36.19 ± 0.17^*a*^	34.13 ± 0.06^*b*^	18.5
	Isoleucine	46.72 ± 0.09^*b*^	47.20 ± 0.00^*a*^	47.13 ± 0.17^*ab*^	46.78 ± 0.13^*ab*^	58.9
	Leucine	80.99 ± 0.17^*d*^	84.49 ± 0.27^*b*^	87.83 ± 0.20^*a*^	83.43 ± 0.13^*c*^	132
	Methionine	13.39 ± 0.13^*b*^	10.48 ± 0.11^*c*^	8.27 ± 0.07^*d*^	14.21 ± 0.15^*a*^	24.3
	Phenylalanine	54.81 ± 0.10^*c*^	57.53 ± 0.17^*b*^	50.85 ± 0.41^*d*^	68.51 ± 0.25^*a*^	38.0
	Proline	50.18 ± 0.21^*a*^	42.08 ± 0.04^*d*^	44.66 ± 0.12^*b*^	43.04 ± 0.09^*c*^	45.6
	Tryptophan	14.95 ± 0.38^*a*^	9.99 ± 0.25^*bc*^	10.47 ± 0.09^*b*^	9.23 ± 0.20^*c*^	27.2
	Valine	46.41 ± 0.60^*b*^	48.79 ± 0.08^*a*^	48.94 ± 0.19^*a*^	45.07 ± 0.15^*c*^	50.3
	Total	395.45 ± 0.58^*a*^	386.01 ± 0.41^*b*^	381.71 ± 0.1.19^*c*^	395.34 ± 0.54^*a*^	480
Ratio hydrophobic : hydrophilic		0.65:1^*a*^	0.63:1^*b*^	0.62:1^*c*^	0.65:1^*a*^	0.81:1
Hydrophobic index^2^		14.73 ± 0.05^*b*^	14.81 ± 0.26^*b*^	14.32 ± 0.12^*c*^	15.23 ± 0.07^*a*^	22.43
Nitrogen conversion factor		5.83 ± 0.00^*a*^	5.73 ± 0.00^*b*^	5.67 ± 0.00^*c*^	5.72 ± 0.00^*b*^	
Nitrogen content (g/100 g dw)		14.28 ± 0.48^*a*^	13.46 ± 0.05^*a*^	14.18 ± 0.53^*a*^	11.72 ± 0.32^*b*^	
Protein content (g/100 g dw)		83.25 ± 2.77^*a*^	77.11 ± 0.29^*b*^	80.42 ± 2.99^*ab*^	67.03 ± 1.83^*c*^	89.7
pH		7.1 ± 0.01^*c*^	7.5 ± 0.01^*a*^	6.4 ± 0.02^*e*^	6.6 ± 0.01^*d*^	7.1 ± 0.02^*b*^
Solubility (gg^−1^)		0.59 ± 0.01^*b*^	0.41 ± 0.03^*c*^	0.12 ± 0.01^*d*^	0.12 ± 0.00^*d*^	1.02 ± 0.00^*a*^

1 Amino acid composition taken from [2]

2 Hydrophobic index per amino acid taken from [20]

### 4.2 Influence of protein concentration on flavor retention

**Figure 2:**
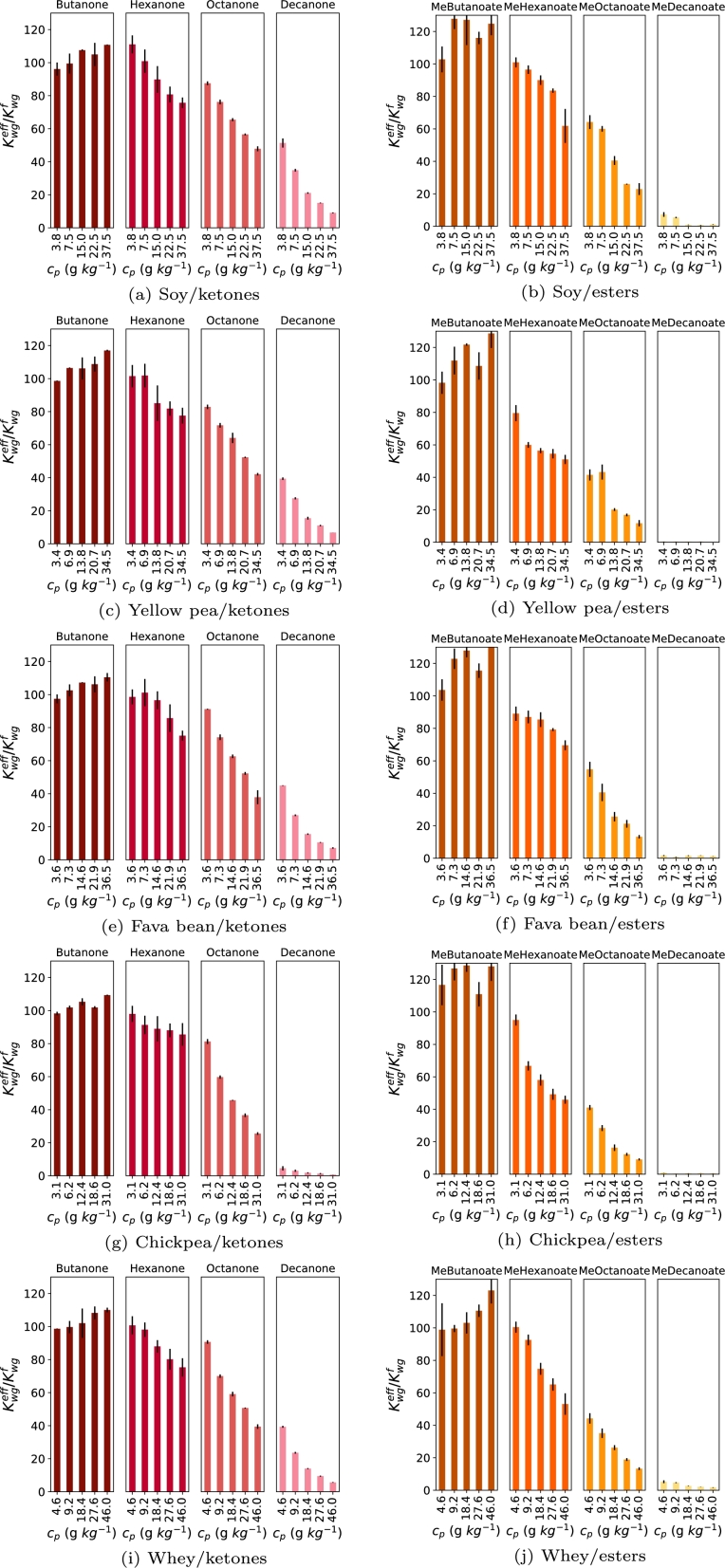
$K_{wg}^{eff}/K_{wg}^{f}$ (peak area flavor in dispersion/peak area flavor in water) measured with APCI-TOF-MS of ketones (a, c, e, g, red), and esters (b, d, f, h, orange) as a function of SPI (a, b), PPI (c, d), FBPI (e, f), CPPI (g,h), and WPI (i,j) concentration. Protein concentration, *c*_*p*_ is the concentration of the isolate corrected for its protein and moisture content. Black bars represent the standard deviation, n = 3.

Headspace concentrations of flavor-protein dispersions were measured with APCI-MS. Four different plant proteins, SPI, PPI, FBPI, and CPPI, and WPPI were analyzed in 5 different concentrations (5-50 gkg^−1^). Protein concentrations were corrected with the known protein and moisture content of each protein isolate. This led to protein concentrations of 3.1-4.6 gkg^−1^ to 31-46 gkg^−1^, depending on the protein isolate. To the protein dispersions, ketones or esters were added and left to equilibrate. The RHC of ketones and esters was measured as a function of plant-protein and whey protein concentration (Fig. 2). A decrease in RHC is interpreted as an increase in flavor retention, as described by eq (12).

*Ketones*: The RHC of butanone significanlty increased with increasing protein content. This suggests a pushing out effect. The pushing out effect of flavors with a short chain length was reported previously for WPI and caseinate with small (hydrophilic) compounds and was attributed to a size-exclusion effect [23]. The size-exclusion effect arises due to steric hindrance caused by large proteins, expelling the small flavor compounds from the dispersion and pushing them into the headspace. For hexanone, octanone and decanone a decrease in RHC was observed with increased protein concentration and log P of the ketone. The exact RHC was compared between the proteins, with lower RHC corresponding to higher retention. With this comparison, flavor retention by the proteins followed CPPI (Fig. 2g) > PPI (Fig. 2c) > FBPI / WPI (Fig. 2e, i) > SPI (Fig. 2a). A small part of hexanone was retained and RHCs hardly decreased (from 96 - 100% to 93 - 100%). Octanone was more retained, evidenced by a decrease in RHCs from 90% to 40%. Decanone was the most retained, and RHCs decreased from 4.5-51% to 0.7-9.1% for the proteins tested.

*Esters*: The RHC of methyl butanoate increased and was significantly higher for the 50 gkg^−1^ dispersion compared to the 5 gkg^−1^ dispersion for all proteins except SPI (Fig. 2b). Similar to butanone, this could be caused by a pushing-out effect [23]. For the other esters, we observed an increase in flavor retention with increased protein content and log P of the esters. Furthermore, RHCs of esters were lower compared to the ketones. The comparison of the exact values of RHC showed that Flavor retention followed CPPI (Fig. 2h) > PPI (Fig. 2d) > FBPI /WPI (Fig. 2f, j) > SPI (Fig. 2b). RHCs of methyl hexanoate decreased from around 100% to 60%. Methyl octanoate decreased from 40% to 10%. The RHCs of methyl decanoate was very low at 5 gkg^−1^ protein, ranging from 1.0-7.3% and further decreasing to 0.1-1.7% at 50 gkg^−1^ isolate dispersion for the different proteins.

### 4.3 Flavor partitioning models

**Figure 3:**
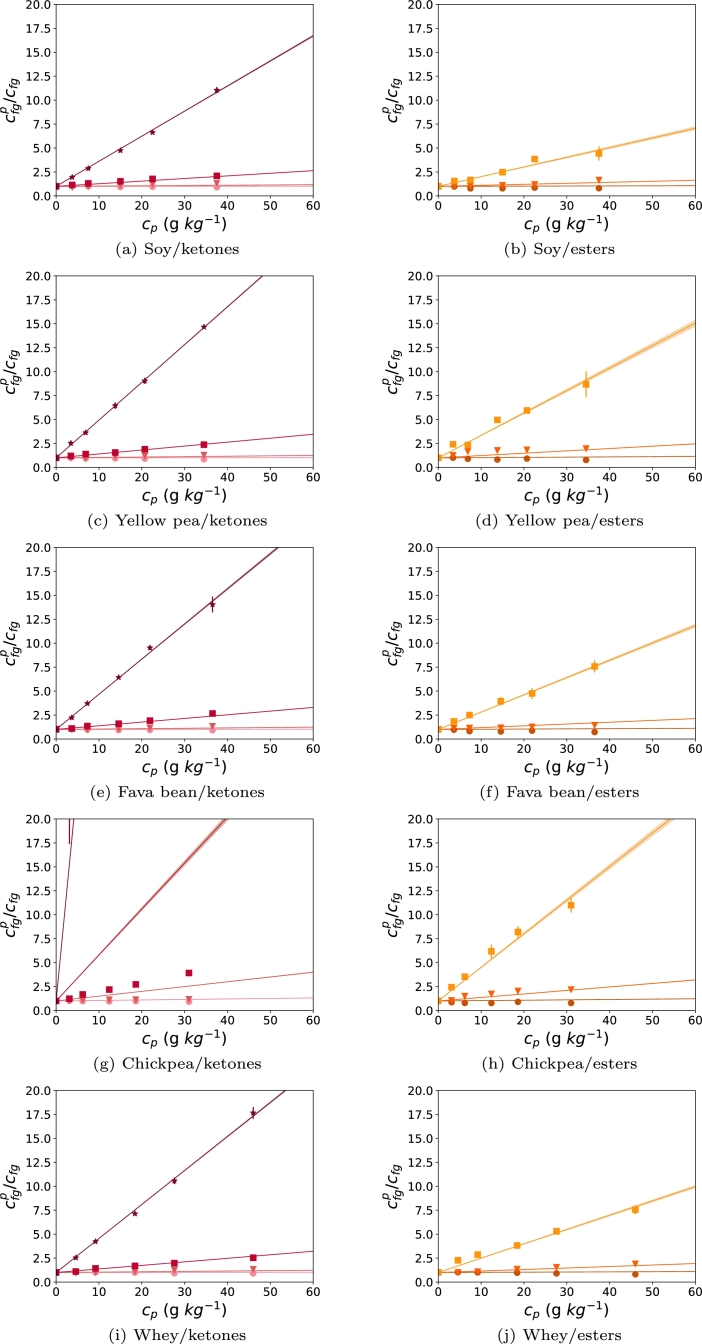
$c_{fg}/c_{fg}^{p}$ (headspace concentration of flavor in water/headspace concentration flavor in dispersion) measured with APCI-TOF-MS and model fits of ketones (a, c, e, g, red), and esters (b, d, f, h, orange) as a function of SPI (a, b), PPI (c, d), FBPI (e, f), CPPI (g,h), and WPI (i,j) concentration. Colored lines represent the model fits of the experimental points for C4 (round), C6 (triangle), C8 (square), and C10 (stars, only ketones). Colored areas around the line represent the uncertainty of the fitted parameter. Colored bars represent the standard deviation, n = 3.

The experimental data were fitted with flavor partitioning models to describe the protein-flavor interactions. The esters and ketones are expected to have hydrophobic interactions and were fitted with eq (14). This resulted in predictions for $a_{p}$, used to describe flavor retention of SPI (Fig. 3a, b), PPI (Fig. 3c, d), FBPI (Fig. 3e, f), CPPI (Fig. 3g, h), and WPI (Fig. 3i, j). Methyl decanoate did not show a linear relation with protein concentration, and was thus not used for the fitting. For the esters more than 93% of the data was predicted with the model and for ketones more than 97% (Table 3). For both esters and ketones, the hydrophobic interaction parameter, $a_{p}$, was highest for CPPI, followed by PPI, FBPI, WPI, and SPI. The esters had lower fitted $a_{p}$ values than the ketones. Interestingly, the $a_{p}$ of CPPI for ketones was 10x higher than the other protein isolates.

**Table 3:** Fitting results of ketones and esters for soy, yellow pea, fava bean, chickpea, and whey protein isolates, using the flavor partitioning model and *P*_*ow*_. Model parameters are the hydrophobic interaction parameter *a*_*p*_ ± uncertainty of the parameter (n=3), and the corresponding residuals squared *R*^2^.

Protein source	Ketones		Esters	
	*a*_*p*_ (10^−5^ Lg^−1^)	*R*^2^	*a*_*p*_ (10^−5^ Lg^−1^)	*R*^2^
Soy	16 ± 0.1	1.00	4.8 ± 0.2	0.93
Yellow pea	25 ± 0.1	1.00	11 ± 0.3	0.96
Fava bean	23 ± 0.2	0.99	8.6 ± 0.2	0.98
Chickpea	290 ± 5.7	0.97	17 ± 0.4	0.97
Whey	22 ± 0.1	1.00	7.2 ± 0.1	0.98

The predictions obtained when flavor compounds were fitted separately are presented in the supplementary material. For these fits, only the protein concentration is varied, while in the fits for the complete dataset, the octanol-water partition coefficients are also considered. From these fits it can be observed that octanone and decanone were closer to the combined $a_{p}$ for ketones than hexanone. The only exception is CPPI, where the $a_{p}$ for decanone was similar to the combined $a_{p}$, which showed that decanone overshadowed the retention of the other ketones. This could also explain the overestimation of hexanone and octanone (Fig. 3g). For the esters, the combined $a_{p}$ was closer to individual $a_{p}$ of methyl hexanoate and methyl octanoate fit values. The poor fit of methyl decanoate confirmed the non linear behavior, except for WPI, which had an $a_{p}$ similar to the overall $a_{p}$ of WPI. Probably, additional effects play a role to explain the very high binding of methyl decanoate. The retention of methyl decanoate was predicted with the obtained fits for $a_{p}$ (supplementary material). All fits underestimated the observed data points.

The parameters obtained in Table 2 were tested for correlation with the $a_{p}$ of the protein isolates. When excluding WPI, a correlation of 0.74 and 0.71 was obtained between the hydrophobic index and $a_{p}$ for ketones and esters, respectively. A correlation of -0.68 and -0.56 was found between solubility and $a_{p}$ (supplementary material).

## 5 Discussion

The headspace concentrations of esters and ketones with different chain lengths were measured at varying protein concentrations. Subsequently, the results were described with a flavor retention model, using the octanol-water partition coefficients of the flavors and fitting a hydrophobic interaction parameter for SPI, PPI, FBPI, CPPI, and WPI. This approach based on one fit parameter per class of flavors (esters or ketones) and protein combination allowed a good description of the protein-flavor interactions. The obtained values could therefore be used as a predictive tool.

An increase in the chain length of flavors resulted in a lower relative headspace concentration, which is in accordance with previous studies on the retention of ketones in PPI, and SPI [25], [9], [12], and for esters in WPI dispersions [23]. The higher flavor retention for longer chain lengths can be explained by the increase in hydrophobicity of these flavors (Table 1). These observations are therefore in line with our expectation and approach for the predictive retention model, in which the octanol-water partition coefficient is included as a variable. This could further explain why a more drastic decrease in headspace concentration is seen for the esters since the octanol-water partition coefficients are 1 log higher (Table 1). Esters were retained around 30% more than the ketones, depending on the flavor compound. However, the model fits showed a higher hydrophobic interaction parameter for ketones. For ketones, the $a_{p}$ was around 2-3 x higher than for esters. For CPPI, $a_{p}$ was even 17x higher for ketones. An ester with a similar log P as the ketone will therefore be less retained.

The model fit parameters make a comparison between the different protein isolates more straightforward. Clearly, the fitted hydrophobic interaction parameter is ordered as follows: CPPI > PPI > FPBI > WPI > SPI. This shows that CPPI and PPI retain more flavor and thus might be harder to flavor, compared to FBPI and SPI. The $a_{p}$ of CPPI was around 10x higher compared to the other proteins for the ketones. The differences between the tested proteins in their ability to retain flavors could be due to different factors. It could be due to the hydrophobic index of the proteins. Although differences between the hydrophobic indexes are small, they were found to be significant (Table 2). A correlation between the hydrophobic index and $a_{p}$ was also found (0.74 and 0.71). A weaker, negative correlation was found with the solubility of protein isolates (-0.68 and -0.56). In other words, a protein isolate that has a lower solubility, or a higher hydrophobic index, is likely to retain more flavor. It has to be noted that apart from protein, other macro- and micro-molecules could be present in the protein isolate that influence flavor retention. Starch, for example, has been shown to retain flavors by entrapment [8]. Furthermore, legumes could contain saponins, that can interact with proteins and form hydrophobic patches that influence flavor retention [12]. In this study, we obtained an $a_{p}$ for the complete ingredients used, which is expected to be dominated by the high protein content.

In this study, retention was explained with hydrophobic interactions only. However, in addition to hydrophobic interactions, other non-covalent interactions could take place as well. By using different disrupting agents, Wang and Arntfield [27] showed that ketones (octanone) are retained with hydrophobic interactions to PPI, while acetate esters formed hydrophobic, electrostatic, and hydrogen interactions. This has also been shown for ethyl esters with myofibrillar protein, where hydrogen bonds proved the most dominant interaction [24]. Therefore, hydrogen bonds are thought to play the dominant role in ester retention. Thus, assuming hydrophobic interactions did explain the results convincingly, most likely because esters with a range of hydrophobicities were selected.

Except for WPI, the retention of methyl decanoate could not be explained with the current model, and was thus excluded. The retention was rapid and a reduction of the headspace of more than 50x was seen for the plant proteins (supplementary material). Possibly, the low solubility of methyl decanoate (8.8 mgkg^−1^) affected the experimental results. For butanone and methyl butanoate, the slope was around 0, which means that these small compounds with low $P_{ow}$ are not retained.

The obtained parameters show that modeling as a function of the octanol-water partition coefficient is sufficient, without distinguishing between the types of non-covalent reactions. Thus, when flavor retention is mostly dependent on hydrophobic interactions and the protein source is known, flavor retention could be predicted.

## 6 Conclusion

In conclusion, the headspace concentrations of esters and ketones in protein dispersions were measured using five different proteins (PPI, SPI, FBPI, CPPI, and WPI). Results showed that flavor retention was explained by protein content, protein type, and hydrophobicity of the flavor compounds. Flavor partitioning models were successfully applied to the different proteins tested. The models assumed mainly hydrophobic interactions for ketones and esters. The obtained hydrophobic interaction parameters for ketones with different proteins were all in the same order of magnitude, except for CPPI. The highest hydrophobic interaction parameters for ketones and esters were fitted for CPPI (290E-05, 17E-5), followed by PPI (25E-05, 11E-5), FBPI (23E-05, 8.6E-5), WPI (22E-05, 7.2E-5) and SPI (16E-05, 4.8E-5). Although the hydrophobic binding constant of CPPI was clearly higher, the differences between the other plant proteins and whey were small. A correlation between the hydrophobic index of the proteins and the hydrophobic binding constant was found. This leads to the conclusion that the retention of esters and ketones is primarily dependent on the flavor compound, and secondary on the protein source.

The assumption of only hydrophobic interactions resulted in good models of the experimental data. The obtained hydrophobic interaction parameter makes it possible to predict flavor-protein interactions for different protein sources to ketones and esters.

## CRediT authorship contribution statement

Silvia J. E. Snel: Conceived an designed the experiments; Performed the experiments; Analyzed and interpreted the data; Wrote the paper. Mirela Pascu, Shane Avison: Performed the experiments; Contributed reagents, materials, analysis tools or data. Igor Bodnár, Atze Jan van der Goot, Michael Beyrer: Conceived and designed the experiments; Contributed reagents, materials, analysis tools or data.

## Declaration of Competing Interest

The authors declare the following financial interests/personal relationships which may be considered as potential competing interests: Michael Beyrer reports financial support and equipment, drugs, or supplies were provided by Firmenich SA.

## Data Availability

Data will be made available on request.

## Appendix A Supplementary material

MMC 1 $c_{fg}/c_{fg}^{p}$ (headspace concentration of flavor in water/headspace concentration flavor in dispersion) measured with APCI-TOF-MS and model fits of esters as a function of SPI (a), PPI (b), FBPI (c), CPPI (d), and WPI (e) concentration. Colored lines represent the model fits of the experimental points for C4 (round), C6 (triangle), C8 (square), and C10 (stars). Methyl decanoate was predicted with fit parameter obtained with C4, C6, and C8. Colored areas around the line represent the uncertainty of the fitted parameter. Colored bars represent the standard deviation, n = 3. MMC 1

MMC 2 Correlation between hydrophobic index, solubility, pH, and amino acid content of pea, soy, fava bean, and chickpea and their model parameters obtained from fitting the flavor retention model on experimental data. The model parameter is the hydrophobic interaction parameter (ap) for esters and ketones. MMC 2

MMC 3 Fitting results of esters and ketones for soy, yellow pea, fava bean, chickpea, and whey protein isolates, using the flavor partitioning model and *P*_*ow*_. Model parameters are the hydrophobic interaction parameter a_*p*,_ the corresponding residuals squared R^2^, and the uncertainty of the parameter a_*p*_. MMC 3
