# Supplementary material for: Flavor-protein interactions for four plant proteins with ketones and esters
Source: Heliyon. 2023 May 25;9(6):e16503. doi: 10.1016/j.heliyon.2023.e16503 (PMC10245154; doi:10.1016/j.heliyon.2023.e16503)
Supplement: MMC 1 — cfg/cfgp (headspace concentration of flavor in water/headspace concentration flavor in dispersion) measured with APCI-TOF-MS and model fits of esters as a function of SPI (a), PPI (b), FBPI (c), CPPI (d), and WPI (e) concentration. Colored lines represent the model fits of the experimental points for C4 (round), C6 (triangle), C8 (square), and C10 (stars). Methyl decanoate was predicted with fit parameter obtained with C4, C6, and C8. Colored areas around the line represent the uncertainty of the fitted parameter. Colored bars represent the standard deviation, n = 3. [file mmc1.docx]

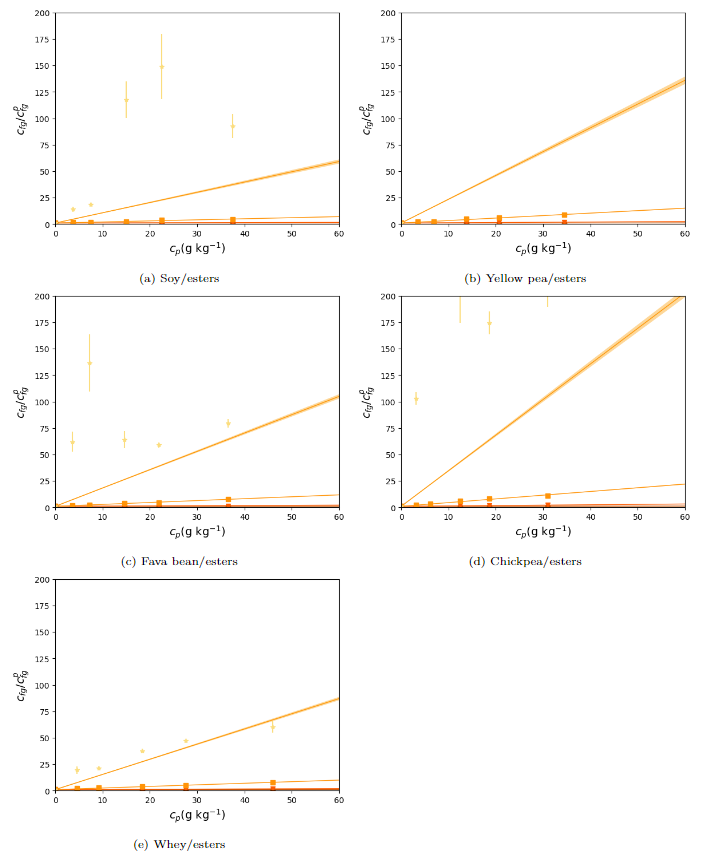


Figure 1: ${c_{fg}}/{c_{fg}^{p}}$(headspace concentration of flavor in water/headspace concentration flavor in dispersion) measured with APCI-TOF-MS and model fits of esters as a function of SPI (a), PPI (b), FBPI (c), CPPI (d), and WPI (e) concentration. Colored lines represent the model fits of the experimental points for C4 (round), C6 (triangle), C8 (square), and C10 (stars). Methyl decanoate was predicted with fit parameter obtained with C4, C6, and C8. Colored areas around the line represent the uncertainty of the fitted parameter. Colored bars represent the standard deviation, n = 3
