# Supplementary material for: Flavor-protein interactions for four plant proteins with ketones and esters
Source: Heliyon. 2023 May 25;9(6):e16503. doi: 10.1016/j.heliyon.2023.e16503 (PMC10245154; doi:10.1016/j.heliyon.2023.e16503)
Supplement: MMC 3 — Fitting results of esters and ketones for soy, yellow pea, fava bean, chickpea, and whey protein isolates, using the flavor partitioning model and Pow. Model parameters are the hydrophobic interaction parameter ap, the corresponding residuals squared R2, and the uncertainty of the parameter ap. [file mmc3.docx]

Table 1: Fitting results of esters and ketones for soy, yellow pea, fava bean, chickpea, and whey protein isolates, using the flavor partitioning model and $P_{ow}$. Model parameters are the hydrophobic interaction parameter a_p,,_ the corresponding residuals squared R^2^, and the uncertainty of the parameter a_p_

| Protein source | Compound | a_p_ (10^-4^Lg^-1^) | R^2^ | Uncertainty a_p_ (10^-4^Lg^-1^) |
| --- | --- | --- | --- | --- |
| SPI | MeButanoate | 0.00 | 0.00 | 2.28E+01 |
|  | MeHexanoate | 0.64 | 0.71 | 1.73E+01 |
|  | MeOctanoate | 0.48 | 0.87 | 5.41E+01 |
|  | MeDecanoate | 1.96 | 0.28 | 5.98E+03 |
| PPI | MeButanoate | 0.00 | 0.00 | 2.04E+01 |
|  | MeHexanoate | 1.64 | 0.00 | 3.80E+01 |
|  | MeOctanoate | 1.12 | 0.91 | 9.85E+01 |
|  | MeDecanoate | 12.67 | 0.46 | 2.71E+04 |
| FBPI | MeButanoate | 0.00 | 0.00 | 2.51E+01 |
|  | MeHexanoate | 0.57 | 0.74 | 8.20E+00 |
|  | MeOctanoate | 0.87 | 0.96 | 5.49E+01 |
|  | MeDecanoate | 1.50 | 0.00 | 7.71E+03 |
| CKPI | MeButanoate | 0.00 | 0.00 | 2.92E+01 |
|  | MeHexanoate | 2.08 | 0.76 | 3.10E+01 |
|  | MeOctanoate | 1.68 | 0.94 | 1.22E+02 |
|  | MeDecanoate | 4.78 | 0.00 | 1.78E+04 |
| WPI | MeButanoate | 0.00 | -0.29 | 1.19E+01 |
|  | MeHexanoate | 0.88 | 0.89 | 1.16E+01 |
|  | MeOctanoate | 0.71 | 0.95 | 4.61E+01 |
|  | MeDecanoate | 0.74 | 0.66 | 9.43E+02 |
| SPI | Butanone | 0.00 | 0.00 | 8.97E+00 |
|  | Hexanone | 4.95 | 0.70 | 1.15E+01 |
|  | Octanone | 1.88 | 0.96 | 9.01E+00 |
|  | Decanone | 1.64 | 1.00 | 2.87E+01 |
| PPI | Butanone | 0.00 | 0.00 | 1.20E+01 |
|  | Hexanone | 5.32 | 0.60 | 1.29E+01 |
|  | Octanone | 2.50 | 0.97 | 1.02E+01 |
|  | Decanone | 2.47 | 1.00 | 2.84E+01 |
| FBPI | Butanone | 0.00 | 0.00 | 8.48E+00 |
|  | Hexanone | 4.56 | 0.68 | 1.05E+01 |
|  | Octanone | 2.66 | 0.95 | 1.65E+01 |
|  | Decanone | 2.30 | 0.99 | 5.56E+01 |
| CKPI | Butanone | 0.00 | 0.00 | 7.29E+00 |
|  | Hexanone | 3.93 | 0.20 | 1.11E+01 |
|  | Octanone | 5.67 | 0.99 | 1.15E+01 |
|  | Decanone | 29.16 | 0.95 | 1.62E+03 |
| WPI | Butanone | 0.00 | 0.00 | 6.82E+00 |
|  | Hexanone | 4.34 | 0.77 | 7.17E+00 |
|  | Octanone | 2.08 | 0.98 | 7.80E+00 |
|  | Decanone | 2.23 | 1.00 | 3.84E+01 |
